# Harlequin frog tadpoles—comparative buccopharyngeal morphology in the gastromyzophorous tadpoles of the genus *Atelopus* (Amphibia, Anura, Bufonidae), with discussion on the phylogenetic and evolutionary implication of characters

**DOI:** 10.1007/s00114-024-01889-6

**Published:** 2024-01-22

**Authors:** Pedro Henrique dos Santos Dias, Marvin Anganoy-Criollo

**Affiliations:** 1 Leibniz Institut Zur Analyse Des Biodiversitätswandels, Zoologisches Museum Hamburg, Zentrum Für Taxonomie Und Morphologie, Martin-Luther-King-Platz 3, 20146 Hamburg, Germany; 2 Departamento de Zoologia, Instituto de Biociências, Universidade de São Paulo, Rua Do Matão No. 101, São Paulo, SP CEP 05508-090 Brazil

**Keywords:** *Atelopus balios*, *A. carrikeri*, *A. nahumae*, *A. nanay*, *A. subornatus*, Systematics

## Abstract

The Neotropical genus *Atelopus* is the most diverse genus of bufonids comprising 99 species. Tadpoles of these frogs are readily distinguished based on the presence of a belly sucker, used by them to stay attached to rocks in fast-flowing streams. Despite their intriguing biology, information about their anatomy is scarce and many morphological systems are unknown. We describe the buccopharyngeal cavity of five *Atelopus* species. The *Atelopus* buccopharyngeal cavity is characterized by (1) presence of a pendulum-like papillae in the prenarial arena, (2) presence of a glandular zone in the prenarial arena, (3) narial vacuities, (4) conical median ridge, (5) absence of buccal roof arena papillae, (6) absence of buccal roof pustulations, (7) single pair of infralabial papillae, (8) absence of lingual papillae, and (9) absence of pustulations in the buccal floor. We propose that characters 1, 2, and 3 are new synapomorphies for the genus. We also propose that the presence of a single pair of infralabial papillae is a synapomorphy for bufonid. Finally, we discuss the convergent evolution of gastromyzophorous and suctorial tadpoles withing anurans.

## Introduction

The Neotropical genus *Atelopus* currently comprises 99 recognized species—the most diverse genus of bufonids, and several other species have been identified and are awaiting a formal description. At least 131 spices (Lötters et al. 2023) are distributed in Central and South Americas, from Costa Rica to Bolivia, along the Andes, Amazonia, and Guiana Shield, from the sea level to elevations up to 3.600 m.a.s.l. (Frost 2023). Contrasting with its large diversity, *Atelopus* is one of the most threatened amphibian genera; the last 30 years witnessed an unprecedent populational decline and many species are considered to be extinct (La Marca et al. 2005; Stuart et al. 2008; Wake and Vredenburg 2008; Lötters et al. 2023).

**Fig. 1:**
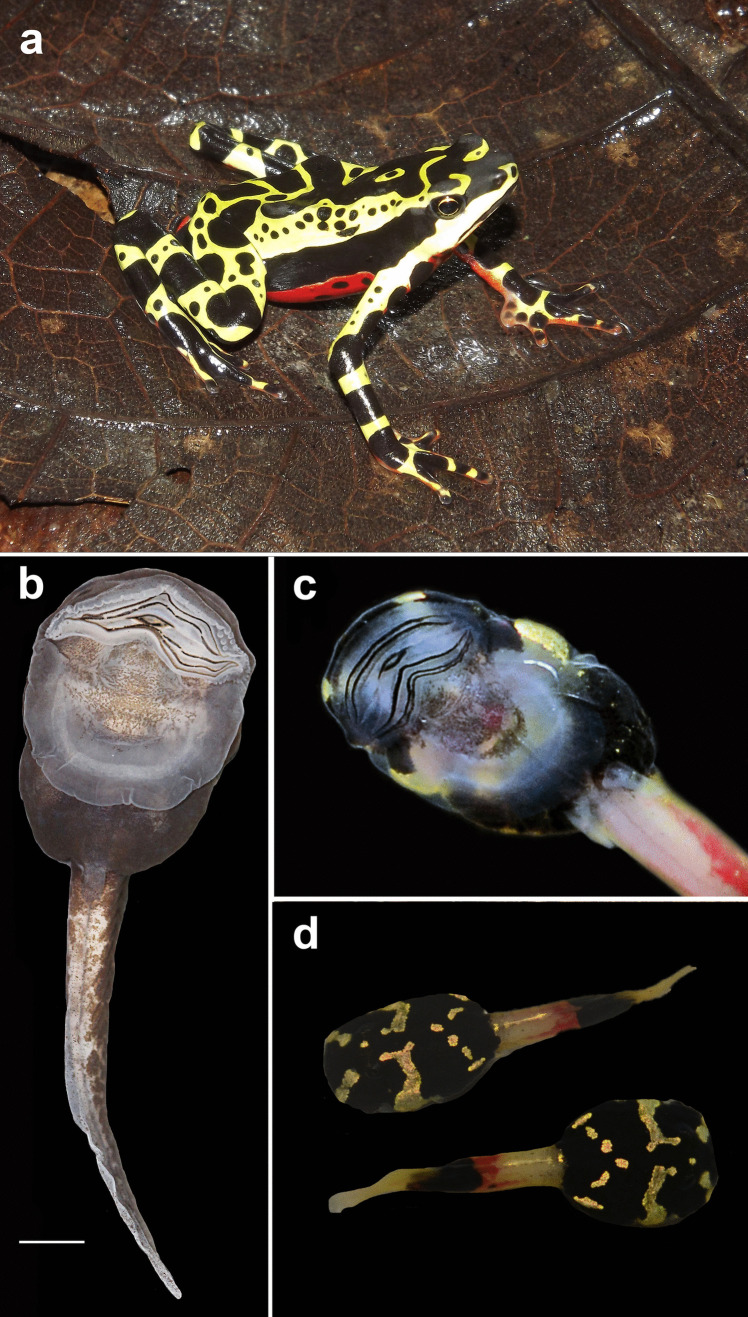
Characteristics of Atelopus. In life (**a**, **c**, **d**), some adults (*Atelopus* sp.) and larvae (*Atelopus subornatus*) have bright coloration. Tadpoles of *Atelopus* are characterized by the presence of a belly sucker (**b**), used by these larvae to attach on rocks. Scale bar = 10 mm

These diurnal, slow-moving frogs are frequently found in association with fast-flowing streams (Lötters 1996). They are popularly known as harlequin frogs due to the bright coloration of many species (Fig. 1a). Also, several species are known to possess tetrodotoxin (TTX) in their skin (Daly et al. 1994; Mebs et al. 1995; Yotsu-Yamashita and Takei 2010), and other compounds have also been reported in *Atelopus* species (see Pearson and Tarvin 2022). *Atelopus* frogs are characterized by their heads longer than broader, bearing a long, acuminate snout (McDiarmid 1971; Peters 1973), interdigital webbing well-developed, and by a reduction in size of the first digit that is often associated with the reduction in the number of phalanges (McDiarmid 1971; Lynch 1993; Fig. 1a). The middle ear is lacking in most species (McDiarmid 1971; Cannatella 1981; Lötters et al. 2011; Pereyra et al. 2016), although *Atelopus* may hear high frequencies, above 1500 Hz, a unique feature among bufonids (Womack et al. 2018).

During breeding season, amplectant pairs can be found in streams in which strings of eggs are laid submerged, beneath rocks and vegetation (Lynch 1986; Lötters 1996; Karraker et al. 2006). The harlequin frog tadpoles may also have bright colors, are gastromyzophorous (Fig. 1), and adapted to live in fast-flowing waters (Altig and Johnston 1989), in which they use their abdominal sucker to attach to rocks (Starrett 1967; Duellman and Lynch 1969; Lynch 1986; Lötters 1996). Tadpoles of 30 species have been described so far (Table 1), but aspects of their internal morphology are restricted to the cranial anatomy of *A. tricolor* (Lavilla and de Sá 2001; Haas 2003). Herein, we describe for the first time the buccopharyngeal morphology for five *Atelopus* species (*A. balios*, *A. carrikeri*, *A. nahumae*, *A. nanay*, and *A. subornatus*) and discuss the evolutionary and phylogenetic implications of our findings for the systematics of bufonids.

**Table 1:** Described tadpoles of *Atelopus*

Species	Reference
*Atelopus balios*	Coloma and Lötters (1996)
*Atelopus carbonerensis*	Mijares-Urrutia and La Marca (2005)
*Atelopus carrikeri*	Rueda-Solano et al. (2015)
*Atelopus certus*	Duellman and Lynch (1969)
*Atelopus cruciger*	Mebs (1980)
*Atelopus ebenoides*	Lynch and Suárez-Mayorga (2011)
*Atelopus elegans*	Marcillo-Lara et al. (2020)
*Atelopus exiguus*	Coloma et al. (2000)
*Atelopus flavescens*	Lescure (1981)
*Atelopus franciscanus*	Boistel et al. (2005)
*Atelopus hoogmoedi*	Gawor et al. (2012); Lötters et al. (2022)
*Atelopus ignescens*	Duellman and Lynch (1969)
*Atelopus laetissimus*	Pérez-Gonzalez et al. (2020)
*Atelopus manauensis*	Gascon (1989)
*Atelopus mindoensis*	Lötters (2001)
*Atelopus mittermeieri*	Acosta-Galvis et al. (2006)
*Atelopus mucubajiensis*	Mijares-Urrutia and La Marca (2005)
*Atelopus nahumae*	Pérez-Gonzalez et al. (2020)
*Atelopus nanay*	Coloma (2002)
*Atelopus palmatus*	Marcillo-Lara et al. (2020)
*Atelopus peruensis*	Gray and Cannatella (1985)
*Atelopus pulcher*	Lötters et al. (2002)
*Atelopus sorianoi*	Mijares-Urrutia and La Marca (2005)
*Atelopus* sp. aff. *spumarius* (Puyo, Ecuador)	Duellman and Lynch (1969)
*Atelopus spumarius*	Rodriguez and Duellman (1994)
*Atelopus subornatus*	Lynch 1986; Enciso-Calle et al. (2017)
*Atelopus tamaense*	Mijares-Urrutia and La Marca (2005)
*Atelopus tricolor*	Lavilla et al. (1997)
*Atelopus varius*	Starrett (1967); Savage (2002)
*Atelopus zeteki*	Lindquist and Hetherington (1998)

## Material and methods

### Buccopharyngeal morphology assessment

We studied the buccopharyngeal morphology in the tadpoles of five species of *Atelopus*. This material is housed at the Instituto de Ciencias Naturales, Universidad Nacional de Colombia (ICN), Bogotá, Colombia, herpetological collection of the Universidad del Magdalena (CBUMAG), Santa Marta, Colombia, and Museo de Zoología de la Pontificia Universidad Católica del Ecuador (QCAZ), Quito, Ecuador. Developmental stages are according to Gosner (1960). Additional bufonids were examined for comparison purposes; we also examined other suctorial and/or gastromyzophorous tadpoles to understand the evolution of buccopharyngeal cavity in these guilds. The complete list of examined material and developmental stages is in the Appendix.

Tadpoles of *Atelopus balios* and *A. nanay* used in the present study are part of the lots used in the tadpoles’ original descriptions: Coloma and Lötters (1996) and Coloma (2002), respectively. Tadpoles of *A. carrikeri* were collected in the same locality as those used in the original description (Rueda-Solano et al. 2015; see also Pérez-Gonzalez et al. 2020). Tadpoles of *A. nahumae* and *A. subornatus* were identified by comparisons with the original descriptions (Lynch 1986; Enciso-Calle et al. 2017; Pérez-Gonzalez et al. 2020) and by comparisons with fresh collected tadpoles of both species (M.A. personal observation).

Two tadpoles per species were dissected according to Wassersug (1976) to expose the buccopharyngeal cavity and stained with methylene blue solution. After inspection under the stereoscopic microscope, one individual per species was submitted to a protocol for scanning electron microscopy (SEM) as follows: (1) samples were washed in distilled water, (2) put in ethanol 25% for 2 h, (3) put in ethanol 70% for 24 h, (4) put in ethanol 100%: 2 baths of 15 min, 20 min prior to the critical point, (5) critical point dried in carbon dioxide, (6) mounted in the stubs with double face carbon tape, and (7) covered with a thin layer gold. Terminology for buccopharyngeal cavity follows Wassersug (1976, 1980) and Dias et al. (2018a).

### Phylogenetic relationships and character optimization

The monophyly of *Atelopus* is well supported by molecular and phenotypical evidence (e.g., McDiarmid 1971; Lötters et al. 2011; Jetz and Pyron 2018). Unfortunately, of the five species studied by us, only *A. nanay* was included in a phylogenetic analysis. Given that the monophyly of the genus is supported, and some characters are invariable within the five species (see “Results”), we discuss the evolution of characters regarding *Atelopus* and other bufonids and treat apomorphic character states as putative synapomorphies for the genus.

We selected taxa for comparison based on Jetz and Pyron’s (2018) phylogenetic hypothesis that has a dense taxonomic sampling. We personally examined representatives of 11 bufonid genera and complemented our dataset with literature information (e.g., Viertel 1982; Müller 2019). The larva of *Frostius erythrophtalmus* is not known, but data is available for *F. pernambucensis* (Dubeux et al. 2023), and we assumed the monophyly of *Frostius* and the sister relationship between *F. erythrophtalmus* and *F. pernambucensis* for optimization purposes. We included representatives of Odontophrynidae larvae, the sister group of Bufonidae in Jetz and Pyron’s (2018) hypothesis, as outgroups. The complete list of examined material and references used is listed in the Appendix.

We propose nine transformation series (Hennig 1966; Grant and Kluge 2004) to account for the variation of the buccopharyngeal morphology in the larvae of *Atelopus* in comparison with other bufonids. The character matrix was built and edited in Mesquite V. 3.70 (Maddison and Maddison 2021) (Supporting Information), and character optimization was performed in T.N.T. v. 1.5 (Goloboff and Catalano 2016). There is no information for several bufonid genera, but we opt to include them in our optimization to demonstrate which parts of the bufonids tree of life require more studies on larval morphology.

## Results

### Buccopharyngeal morphology

The buccopharyngeal morphology of the five species is quite similar. A single, condensed, description is provided and differences noted when present.

#### Buccal roof (Figs. 2a, 4a, 5a, 6a, 7a) triangular

Prenarial arena semi-elliptical, with a pendulum-like papilla (Fig. 3a) and several secretory pits (Fig. 3b, c; absent in *A. subornatus*); these pits are located immediately posterior to the upper jaw sheath and before the pendulum-like papilla, covering the entire width of that region. The pits are rounded, and a secretion residue can be observed in several pits (Fig. 3c). Internal nares elliptical, transversally oriented; posterior valve free, lacking marginal projection. Vacuities (Fig. 3d, e) present, circumscribed by margins of inner nares, presenting ciliated cells (Fig. 3f). Postnarial arena diamond-shaped, two conical, tall postnarial papillae; first pair shorter than second pair. Lateral ridge papillae short, triangular, bifurcated (not bifurcated in *A. carrikeri*). Median ridge low, conical (bifurcated in *A. carrikeri*), papilla-like. Buccal roof arena poorly defined, completely lacking papillae or pustulation. Dorsal velum medially discontinued, devoid of papillae or projections, arch-shaped.

**Fig. 2:**
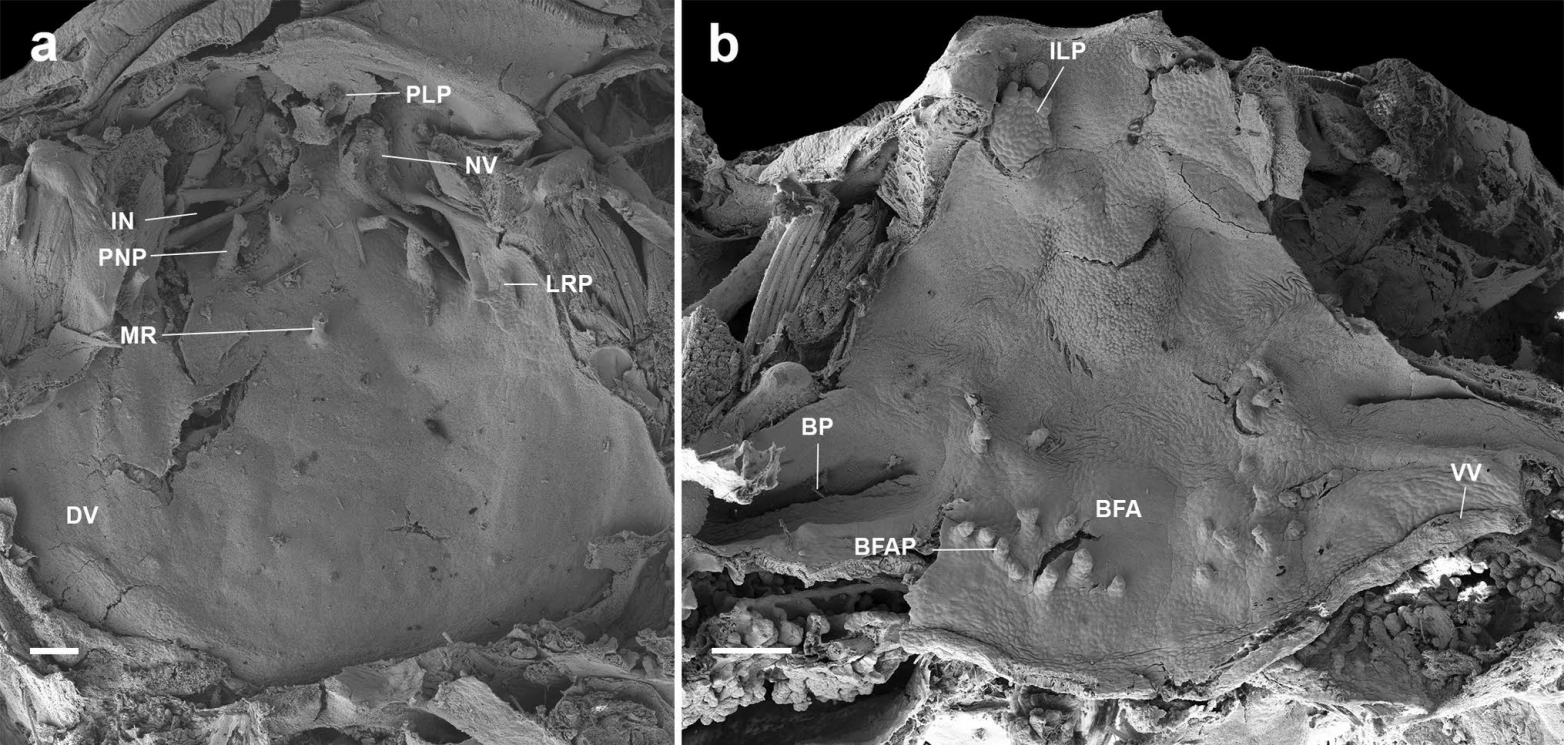
Buccal roof (a) and floor (b) of the tadpole of *Atelopus balios* (QCAZ 2670) at stage 34. BFA, buccal floor arena; BFAP, buccal floor arena papillae; BP, buccal pocket; DV, dorsal velum; ILP, infralabial papillae; IN, internal nares; LRP, lateral ridge papilla; MR, median ridge; NV, naria vacuities; PLP, pendulum-like papillae; PNP, postnarial papillae; VV, ventral velum. Scale bar = 100 µm

**Fig. 3:**
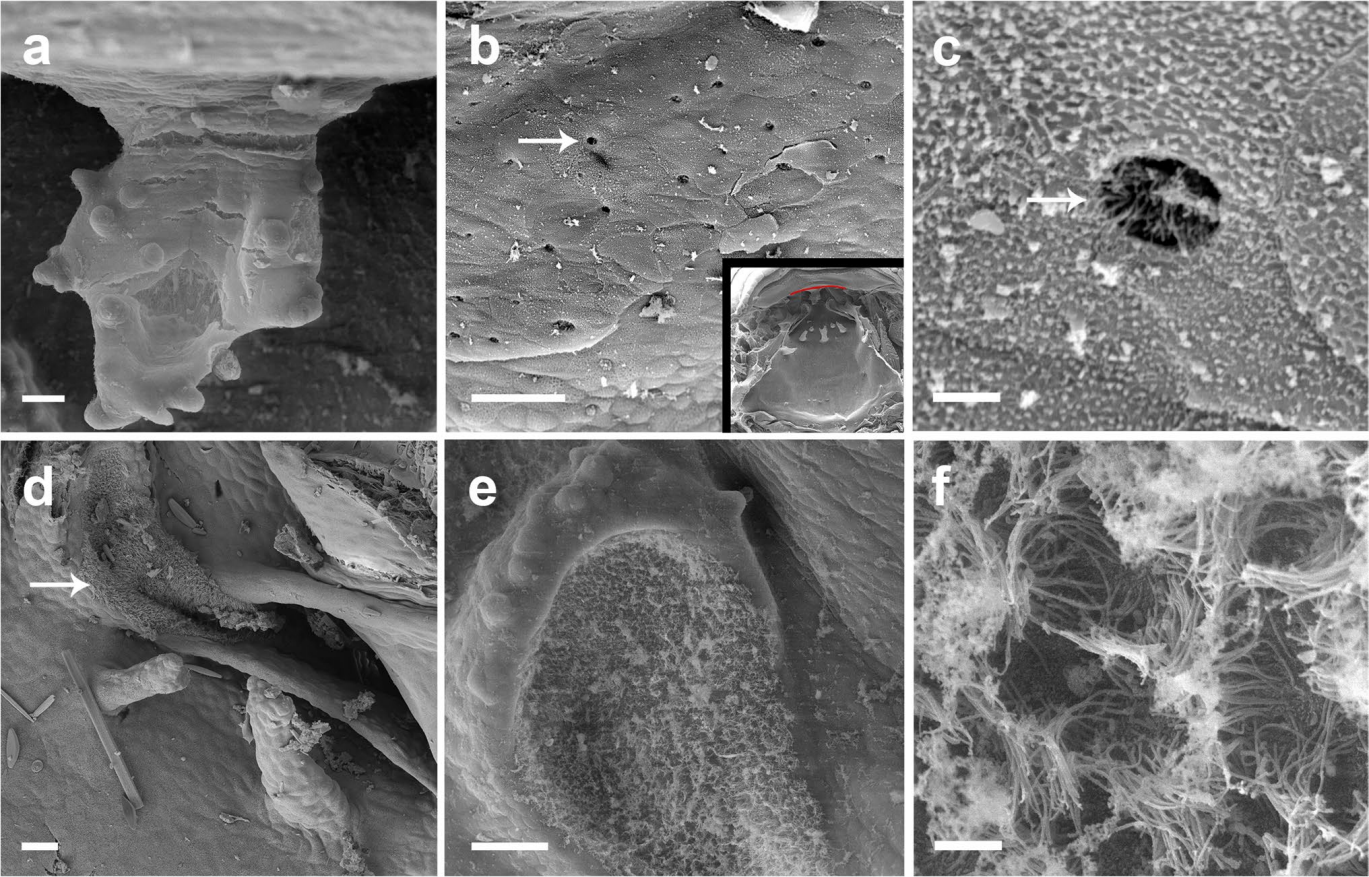
Anatomical details of the pendulum-like papilla in the prenarial arena (**a**), of the glandular zone (**b**, **c**), of the narial vacuities (**d**, **e**) with its ciliated epithelium (**f**) in the larvae of *Atelopus carrikeri* (CBUMAG 0892) at stage 35. Inset in (**c**) showing in red the area in the buccal roof where the secretory pits can be found. Character states, when present, are identical in the other studied species. Scales bars = 50 µm (**a**, **d**, **e**), 20 µm (**b**), and 2 µm (**c**, **f**)

#### Buccal floor (Figs. 2b, 4b, 5b, 6b, and 7b) triangular

Single pair of flat, wide, infralabial papillae; tip crenulated. Lingual bud poorly defined; lingual papillae absent. Buccal floor arena bell-shaped; buccal floor arena papillae present (10–11 in *A. balios*; 10–12 in *A. carrikeri*; 13–14 in *A. nahumae*; 9–11 in *A. nanay*; 7–8 in *A. subornatus*). Buccal floor arena lacking pustulations. Prepocket papillae and pustulation absent. Buccal pockets deep, wide, oblique slit-shaped. Ventral velum present; spicular support inconspicuous; medial notch absent; marginal projections present; secretory pits poorly developed; secretory ridges present. Branchial basket triangular, short, poorly developed, wider than long. Three filter cavities, well-defined, partially covered by ventral velum.

**Fig. 4:**
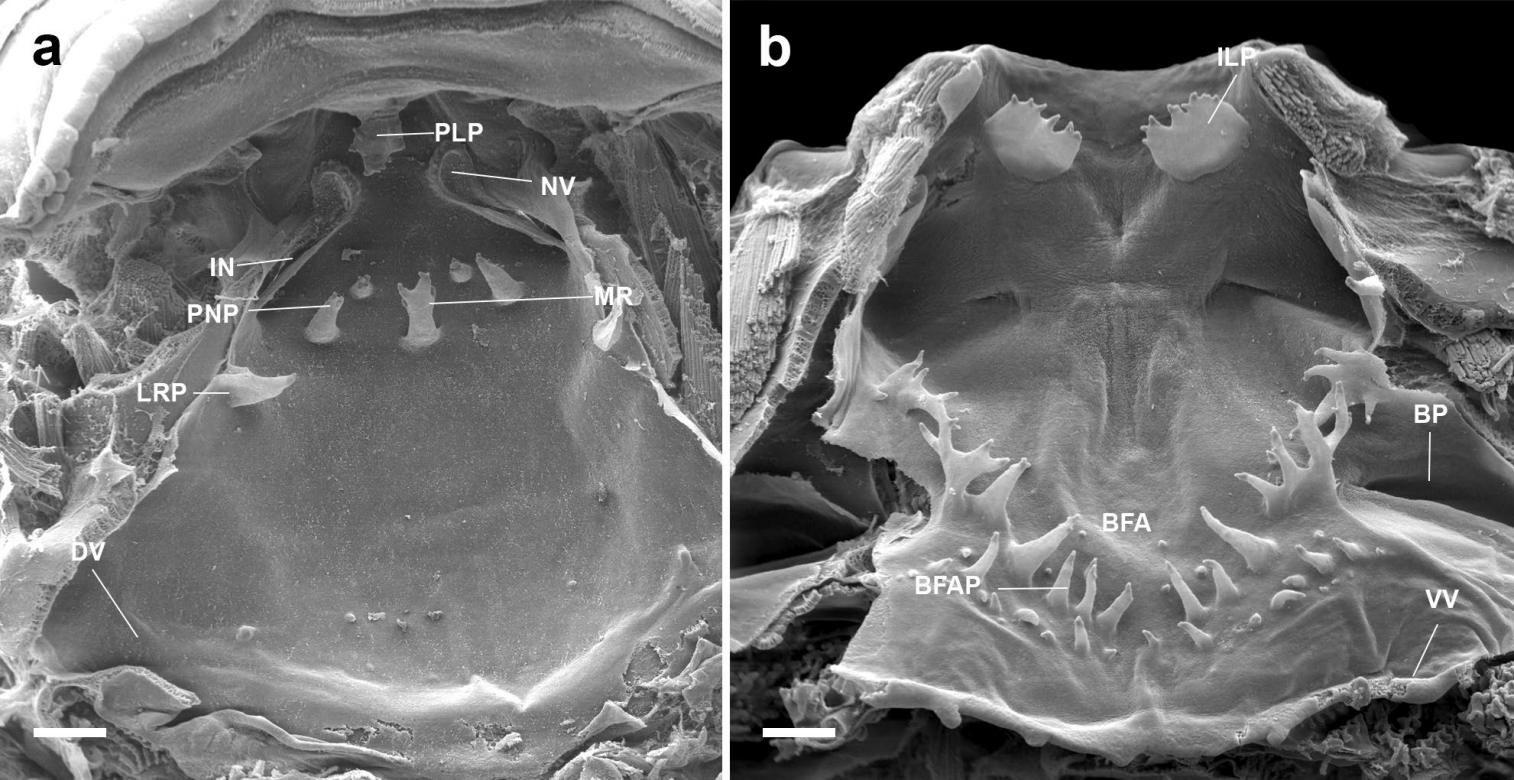
Buccal roof (a) and floor (b) of the tadpole of *Atelopus carrikeri* (CBUMAG 0892) at stage 35. BFA, buccal floor arena; BFAP, buccal floor arena papillae; BP, buccal pocket; DV, dorsal velum; ILP, infralabial papillae; IN, internal nares; LRP, lateral ridge papilla; MR, median ridge; NV, naria vacuities; PLP, pendulum-like papillae; PNP, postnarial papillae; VV, ventral velum. Scale bar = 100 µm

**Fig. 5:**
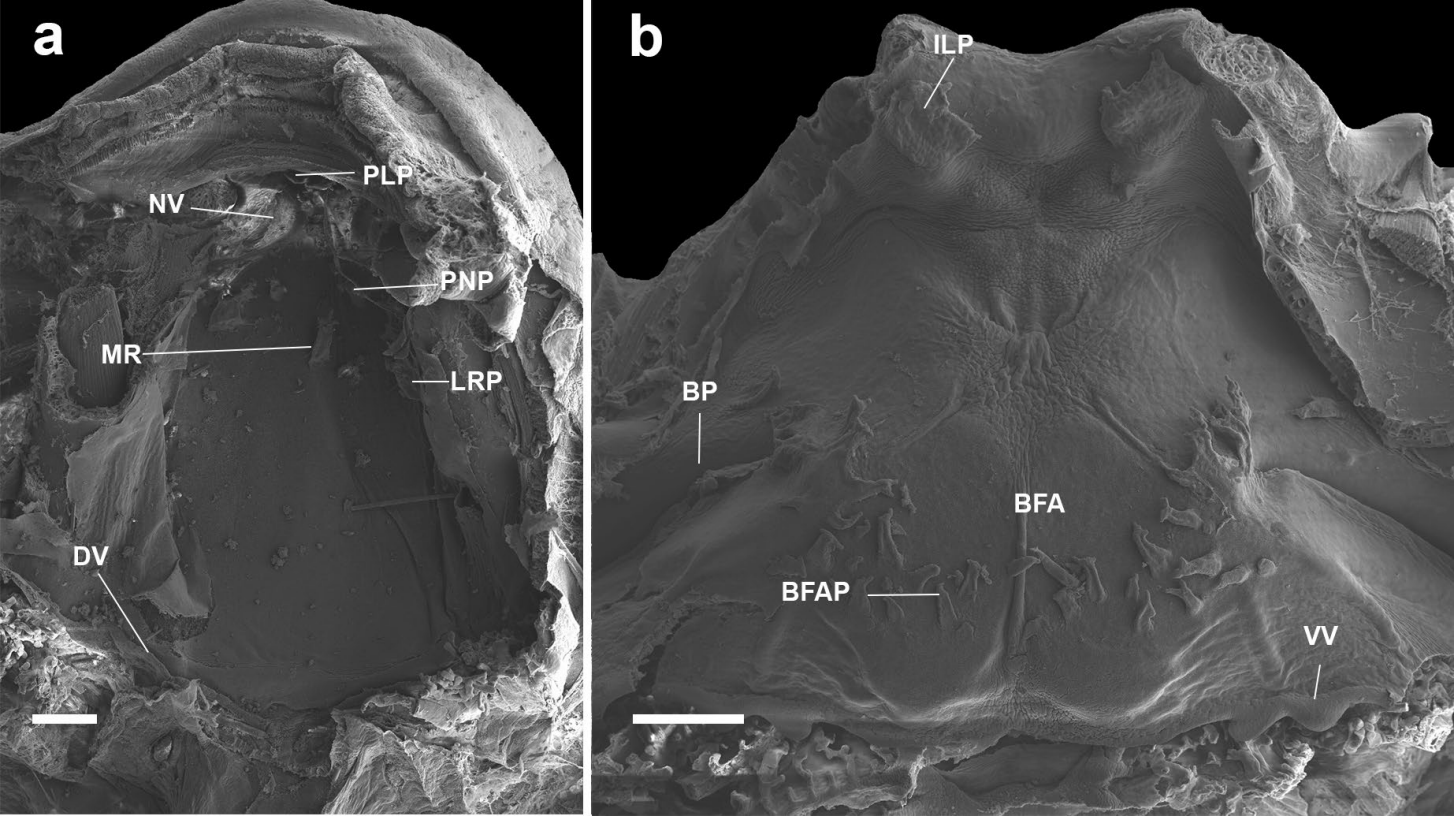
Buccal roof (a) and floor (b) of the tadpole of *Atelopus nahumae* (ICN 33202) at stage 29. BFA, buccal floor arena; BFAP, buccal floor arena papillae; BP, buccal pocket; DV, dorsal velum; ILP, infralabial papillae; IN, internal nares; LRP, lateral ridge papilla; MR, median ridge; NV, naria vacuities; PLP, pendulum-like papillae; PNP, postnarial papillae; VV, ventral velum. Scale bar = 100 µm

**Fig. 6:**
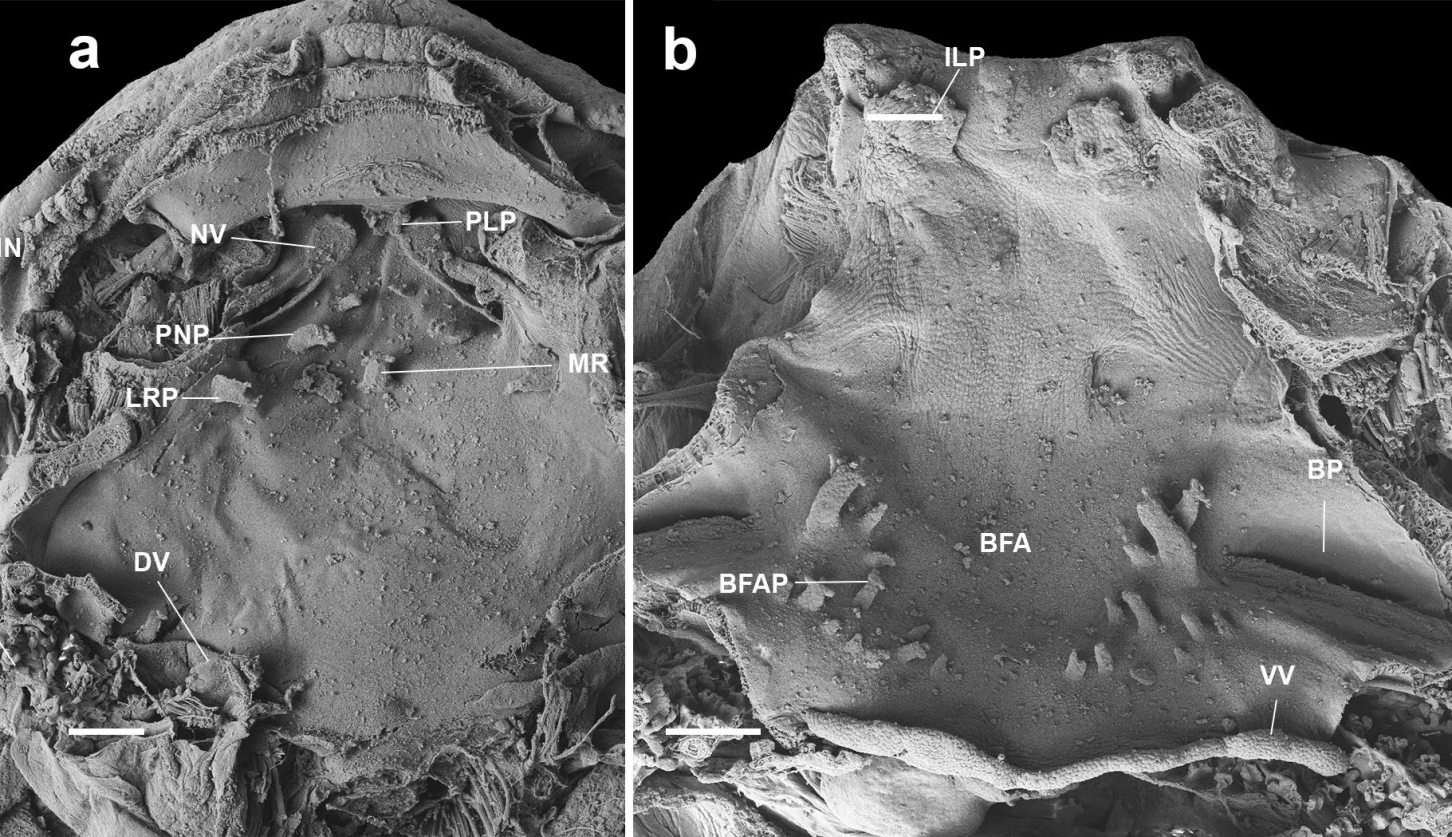
Buccal roof (a) and floor (b) of the tadpole of *Atelopus nanay* (QCAZ 3672) at stage 27. BFA, buccal floor arena; BFAP, buccal floor arena papillae; BP, buccal pocket; DV, dorsal velum; ILP, infralabial papillae; IN, internal nares; LRP, lateral ridge papilla; MR, median ridge; NV, naria vacuities; PLP, pendulum-like papillae; PNP, postnarial papillae; VV, ventral velum. Scale bar = 100 µm

**Fig. 7:**
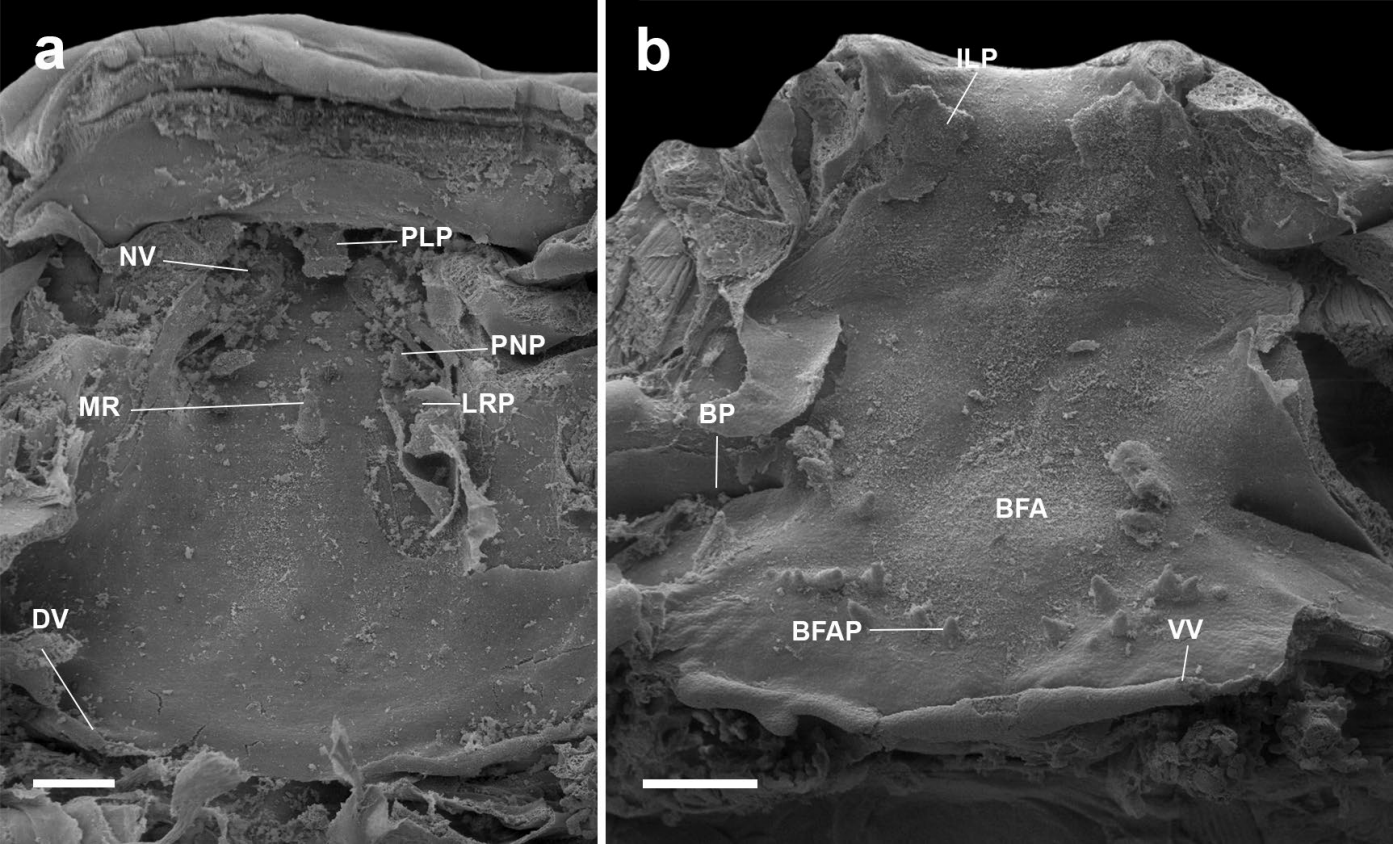
Buccal roof (a) and floor (b) of the tadpole of *Atelopus subornatus* (ICN 31435) at stage 32. BFA, buccal floor arena; BFAP, buccal floor arena papillae; BP, buccal pocket; DV, dorsal velum; ILP, infralabial papillae; IN, internal nares; LRP, lateral ridge papilla; MR, median ridge; NV, naria vacuities; PLP, pendulum-like papillae; PNP, postnarial papillae; VV, ventral velum. Scale bar = 100 µm

### Evolution of characters

#### Character 1: prenarial arena, pendulum-like papilla: absent (0), present (1)

The prenarial arena is the area between the internal nares and the mouth opening (Wassersug 1976). Several structures have been reported in that region in different anuran larvae, such as crests, ridges, and pustulations, among others (e.g., Wassersug 1980; Vera Candioti 2007; Nascimento et al. 2013; Dias et al. 2018a, b). *Atelopus* larvae have a pendulum-like papillae (state 1; Fig. 3a).

##### Taxonomic distribution and optimization

**Fig. 8:**
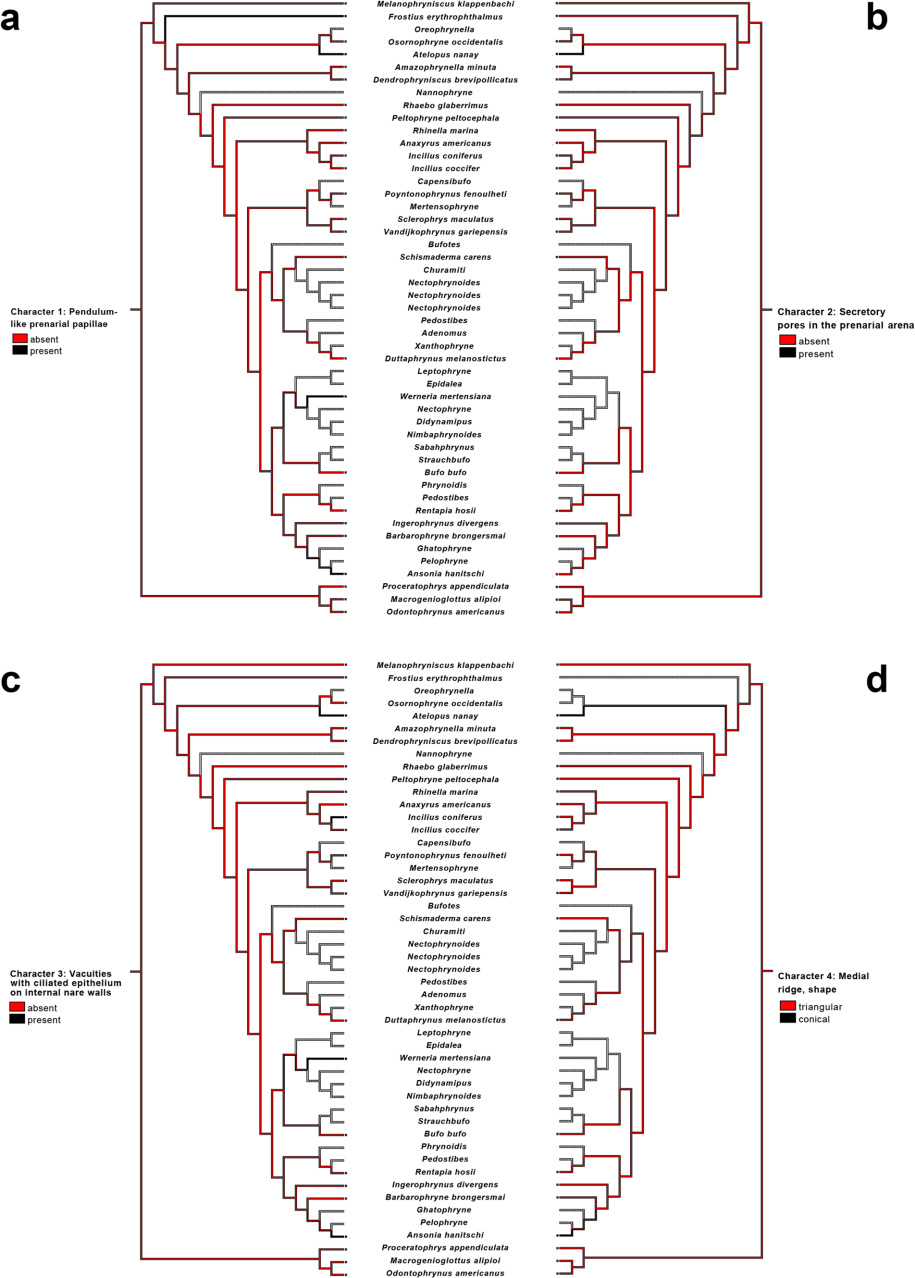
Parsimonious optimization of characters 1 (**a**), 2 (**b**), 3 (**c**), and 4 (**d**). Gray represents unknown condition

**Fig. 9:**
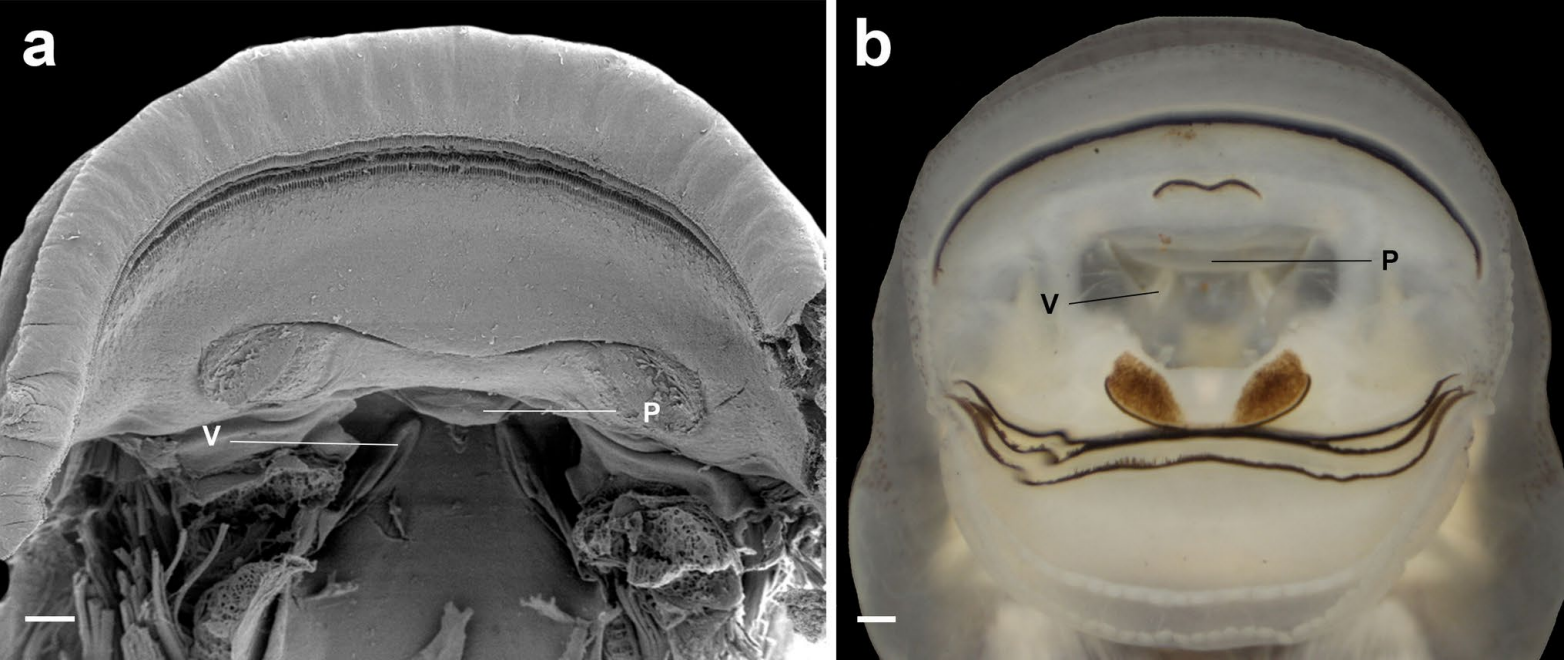
Presence of a pendulum-like papillae and of narial vacuities in the bufonid larvae of *Ansonia hanitschi* (ZMH A08803; stage 29) and *Werneria mertensiana* (ZMB 79695; stage 30). Scale bars = 200 µm

The presence of a pendulum-like papillae was invariable in the five studied *Atelopus* species and also present in *Frostius pernambucensis* larvae; current optimization of this character (Fig. 8a) suggests it as a synapomorphy of *Atelopus*. Additionally, similar papilla was observed in the suctorial tadpoles of *Ansonia* and *Werneria* (Fig. 9), but the absence of data for their close related taxa (*Ansonia*: *Pelophryne* and *Ghatophryne*; *Werneria*: *Nectophryne*, *Didynapius*, and *Nimbaphrynoides*) renders the optimization ambiguous within these lineages.

#### Character 2: prenarial arena, glandular zone: absent (0), present (1)

In the species of *Atelopus*, a large portion of the prenarial arena is covered with small pits (Fig. 3b), very similar to secretory pits of the ventral velum of other tadpoles. These pits are rounded, deep, being secreted. The function of these pits in such peculiar region is unknown.

##### Taxonomic distribution and optimization

Character state 1 was observed only in *Atelopus* (except in *A. subornatus*), and we suggest it as a new synapomorphy for the genus (Fig. 8b). As far as we know, it has not been reported in any other anuran larvae.

#### Character 3: vacuities circumscribed by margins of inner nares: absent (0), present (1)

Van Eeden (1951:9) found what he called “a band of ciliated epithelium” in *Ascaphus truei* and suggested that the cilia could have some role in the feeding mechanism proposed by Noble (1927). Wassersug (1980) described this “*cul de sac*” feature in other taxa and suggested that it may have a chemosensory function. Later, vacuities have been reported in several taxa, particularly in Cophomantinae (e.g., Kolenc et al. 2008), Leptodactylidae (e.g., Nascimento et al. 2021), Centrolenidae (e.g., Rada et al. 2019; Dias et al. 2020), and in tadpoles of the *Scinax perpusillus* species group (e.g., Dias and Pie 2021).

##### Taxonomic distribution and optimization

We observed the presence of vacuities in all the five *Atelopus* examined, which suggested it as another synapomorphy for the genus (Fig. 8c). We also observed the presence of vacuities in *Ansonia* and *Werneria* (Fig. 9); however, as discussed above (see character 1), the lack of data precludes a non-ambiguous optimization of this character state in those clades.

#### Character 4: median ridge, shape: triangular (0), conical (1), trapezoidal (2); unordered

The median ridge marks the end of the postnarial arena. It is a feature highly variable among tadpoles. According to Wassersug (1980), due to its central location, the median ridge may play a role in splitting the respiratory current into right and left ones. The shape of the median ridge has been used as a character in the systematics of several groups; for instance, Dias et al. (2019a) suggested a trapezoidal median ridge as a synapomorphy for the *Proceratophrys bigibbosa* species group. Within examined taxa, three different morphologies were observed for the median ridge: triangular (state 0), as in most of bufonids, conical, as in *Atelopus* and *Ansonia* (state 1), and trapezoidal (state 2) in some outgroup taxa (e.g., *Odontophrynus*).

##### Taxonomic distribution and optimization

Conical medial ridge was present in all *Atelopus* examined and also in *Ansonia*. The absence of median ridge in *Osornophryne* (inapplicable) and the lack of data for *Oreophrynella* render the optimization of this character ambiguous (Fig. 8d). It is likely that *Oreophrynella* will also lack a median ridge due to its endotrophic development—see Wassersug and Duellman (1984) for discussion of buccopharyngeal cavity in endotrophic and direct-developer frogs—which will prevent the optimization of this character in the future as well.

#### Character 5: buccal roof arena papillae: absent (0), present (1)

The buccal roof arena papillae are usually conical, with one or few bifurcated papillae (Wassersug 1976). Some authors (e.g., Wassersug 1980) suggested that these papillae may contribute to the sorting of food particles in the mouth. These papillae delimitate the buccal roof arena and may be very abundant, as in *Hylodes* (e.g., Montesinos et al. 2022), or completely absent, as in *Atelopus*, which renders the buccal roof arena also absent.

##### Taxonomic distribution and optimization

**Fig. 10:**
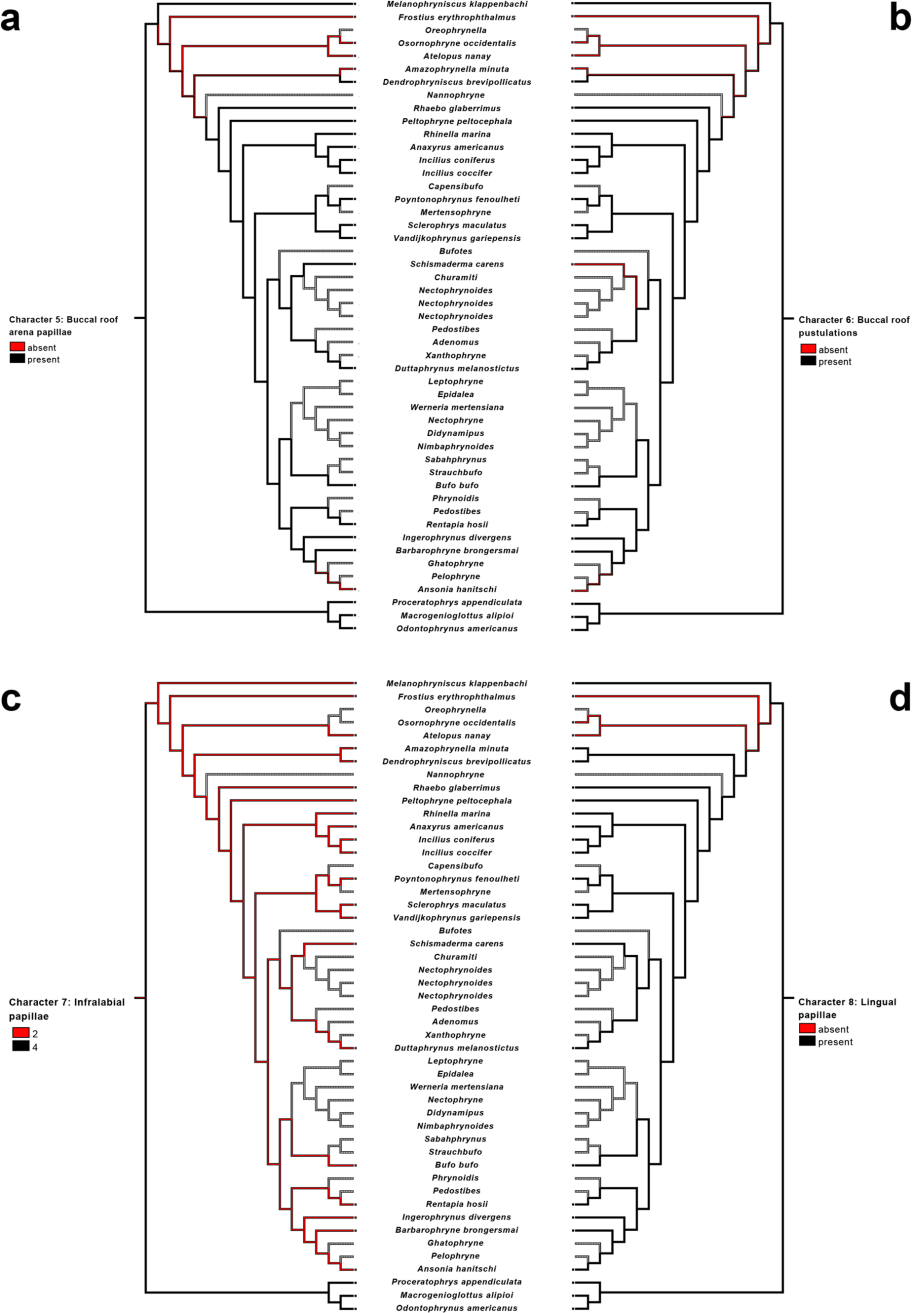
Parsimonious optimization of characters 5 (**a**), 6 (**b**), 7 (**c**), and 8 (**d**). Gray represents unknown condition

The optimization of this character is complex (Fig. 10A); we found the buccal roof arena papillae absent in all examined *Atelopus*, plus in the endotrophic larvae *Frostius pernambucensis*, in the direct developer tadpole-like *Osornophryne occidentalis*, in *Amazophrynella minuta*, and in *Ansonia*. The optimization of this character is ambiguous in all mentioned taxa. The presence of buccal roof papillae in *Dendrophryniscus brevipollicatus* and the absence of data for *Nannophryne* make the optimization of this character difficult at the base of Bufonidae except *Melanophryniscus*.

#### Character 6: buccal roof pustulations: absent (0), present (1)

In many anuran taxa, both buccal floor and roof are covered with a field of rounded pustulation. The function of these structures is unknown, but its abundancy seems to be correlated with benthic, lotic species (e.g., Vera Candioti 2007; Dias et al. 2014). The reduction or absence of pustulations has been reported in endotrophic (e.g., Wassersug and Duellman 1984; Wassersug and Heyer 1988), fossorial (e.g., Wassersug 1980; Rada et al. 2019; Dias et al. 2020), macrophagous (e.g., Wassersug 1980; Vera Candioti et al. 2004; Vera Candioti 2005; Dias et al. 2019b), oophagous (e.g., Vera Candioti et al. 2021), and suctorial (e.g., Wassersug and Heyer 1988) tadpoles. In all examined *Atelopus*, there was no pustulation in the buccal roof.

##### Taxonomic distribution and optimization

The absence of pustulations followed the exact same pattern as that of the absence of buccal roof arena papillae, being absent in *Ansonia*, *Amazophrynella minuta*, *Frostius pernambucensis*, *Osornophryne occidentalis*, and *Schismaderma carens*. Thus, the optimization of this character is highly ambiguous (Fig. 10b).

#### Character 7: number of infralabial papillae: 2 (0), 4 (1)

The infralabial papillae are the first papillae observed in the buccal floor; they are positioned right after the mouth’s opening and can vary in number, size, and shape—there may be a single pair as in *Cycloramphus stejnegeri* (Wassersug and Heyer 1983) or up to 12 in tadpoles of *Heleophryne natalensis* (Wassersug and Heyer 1988); they can be conical (e.g., Vera Candioti 2007) or branched (e.g., Dias et al. 2019a). Wassersug (1980) hypothesized that these papillae play an important role selecting food particles that will enter in the buccal cavity of tadpoles.

##### Taxonomic distribution and optimization

*Atelopus* as well as all other bufonids present only a single pair (two papillae) of infralabial papillae, contrasting with the two pairs (four) of papillae in Odontophrynidae. Thus, the presence of two infralabial papillae is a synapomorphy for Bufonidae (Fig. 10c).

#### Character 8: lingual papillae: absent (0), present (1)

Lingual papillae are located in the tongue anlage (Wassersug 1976) and are likely to have gustatory function (Hammerman and Thomas 1967). Lingual papillae are present in most frogs, although absent by definition in the aglossal pipids and in several other lineages.

##### Taxonomic distribution and optimization

*Atelopus* larvae lack lingual papillae (state 0). The same condition was observed in *Frostius* and *Osornophryne*. Given the presence of lingual papillae in *Melanophryniscus* and other bufonids, the optimization of this character was ambiguous (Fig. 10d).

#### Character 9: buccal floor pustulations: absent (0), present (1)

Pustulations are commonly present in the buccal floor of tadpoles (e.g., Vera Candioti 2007; Nascimento et al. 2013; Dias et al. 2014) and have rarely been reported absent (e.g., *Ascaphus truei*; Wassersug 1980).

##### Taxonomic distribution and optimization

**Fig. 11:**
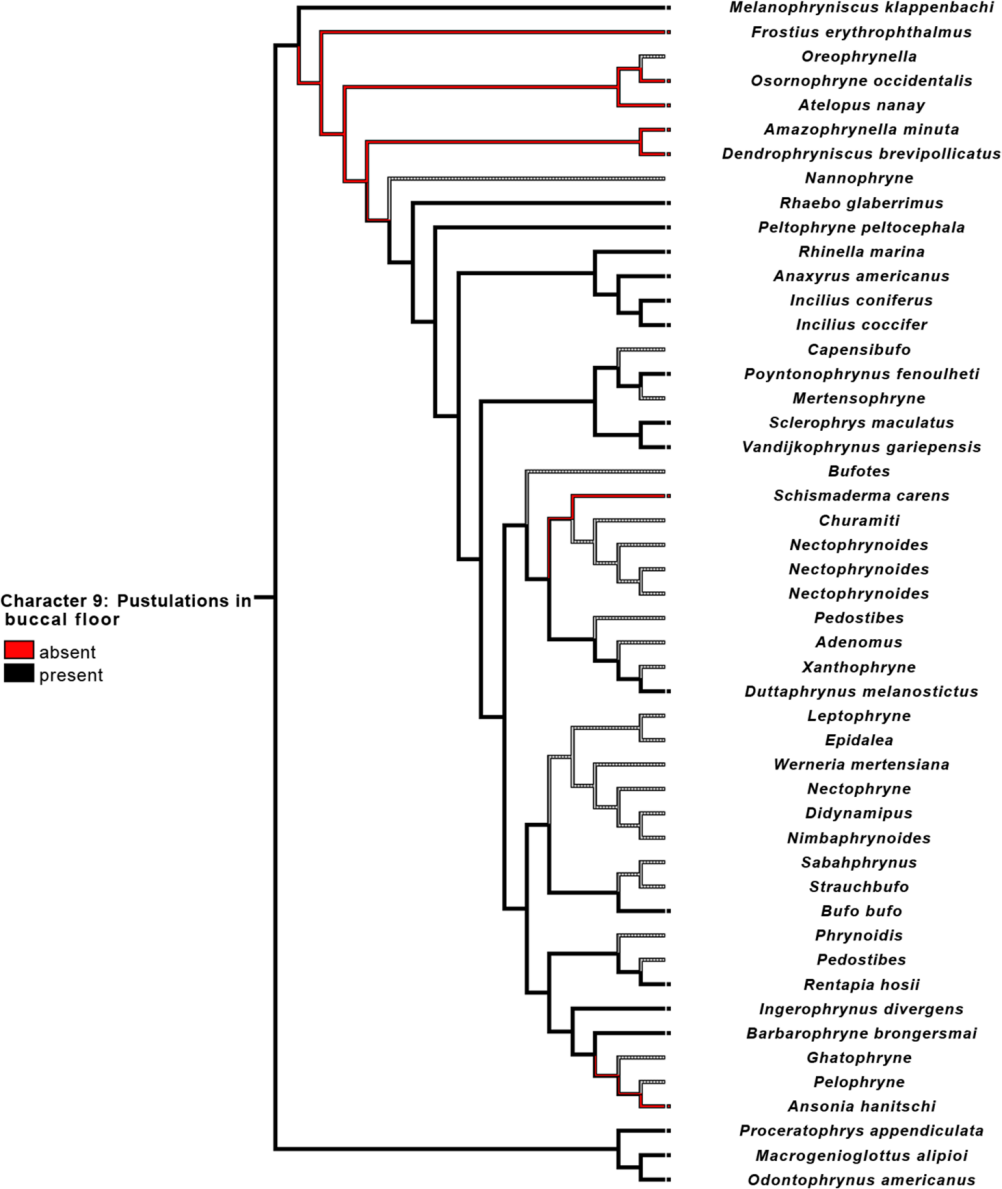
Parsimonious optimization of character 9. Gray represents unknown condition

Pustulations on the buccal floor were absent in all examined *Atelopus*. Also, *Frostius*, *Osornophryne*, *Amazophrynella*, and *Dendrophryniscus* lacked these pustulations, rendering its absence a synapomorphy for all bufonids minus *Melanophryniscus* (Fig. 11). Pustulations were also absent in *Ansonia* and *Schismaderma carens*.

## Discussion

### Larval morphology and the systematics of bufonids

*Atelopus* have a distinctive larva within bufonids; their abdominal sucker, wide oral disc, and color pattern (e.g., Duellman and Lynch 1969; Lötters 1996; Pérez-Gonzalez et al. 2020) make these tadpoles easily distinguished from other bufonids. The comparative analysis of the buccopharyngeal cavity of *Atelopus* revealed a series of new unique, intriguing character states in these tadpoles. The *Atelopus* buccopharyngeal cavity is characterized by (1) presence of a pendulum-like papillae in the prenarial arena, (2) presence of a glandular zone in the prenarial arena, (3) narial vacuities, (4) conical median ridge, (5) absence of buccal roof arena papillae, (6) absence of buccal roof pustulations, (7) single pair of infralabial papillae, (8) absence of lingual papillae, and (9) absence of pustulations in the buccal floor. We propose that characters 1, 2, and 3 are new synapomorphies for the genus *Atelopus*.

The prenarial arena of tadpoles is characterized by the presence of several features, ranging from pustulations to crests (e.g., Vera Candioti 2007). These features play an important role in the feeding mechanism of tadpoles; for instance, it has been hypothesized that the presence of an inverted V structure in the prenarial arena of umbelliform tadpoles’ interlocks with the infralabial papillae to prevent large food particles from entering the mouth (Wassersug 1980; Dias et al. 2018b, 2021). The presence of a large, pendulum-like papillae in the larvae of *Atelopus* is very intriguing; a similar feature is rare among anurans. We speculate two putative functions to it: (1) such large papilla would prevent large particles of reaching the vacuities’ area; (2) it could deviate the water flow to the vacuities. It is important to note that vacuities possibly have a chemosensory function (Wassersug 1980), and both diverting water towards them, as well as preventing them from being obstructed by large particles, seem possible explanations for the presence of this pendulum-like papillae. Nevertheless, further studies are required to test our hypotheses. Interesting to note that a similar, although narrower, feature was described in the prenarial arena of *Rhinella quechua* (Aguayo et al. 2009), another gastromyzophorous bufonid.

The posterior region of buccal floor is usually marked by secretory tissue in most anurans (Kenny 1969; Wassersug 1980)—secretory tissue, however, may be present in other regions, as in the buccal floor of *Rhacophorus vampyrus* (Vera Candioti et al. 2021). Usually, secretory cells are organized in pits and ridges (Wassersug and Rosenberg 1979). Many authors agree with the hypothesis that the secretory pits, through the production of mucus strands, may aid in food entrapment (de Jongh 1968; Kenny 1969; Wassersug 1976, 1980; Wassersug and Rosenberg 1979). Although experimental studies testing this hypothesis are lacking, the anatomy and topographical distribution of the secretory pits provide some support to that view. We observed secretory pits in the prenarial arena the *Atelopus* tadpoles examined, except in *A. subornatus* that phenotypically resembles the secretory pits of the velum of anuran larvae. The function of these pits is unknown and, as far as we know, this character state has never been reported for other anurans. Further studies are required to understand the biological meaning of these pits, notwithstanding, and we propose their presence in the prenarial arena as a synapomorphy for the genus *Atelopus*.

Vacuities were originally described in *Ascaphus* tadpoles (van Eeden 1951) and since then reported in few taxa (e.g., Wassersug 1980; d’Heursel and Haddad 2007; Magalhães et al. 2015; Pezzuti et al. 2015; Dias and Pie 2021). Kolenc et al. (2008) suggested that the presence of vacuities was a synapomorphy for the Cophomantini tribe. Dias et al. (2020; see also Rada et al. 2019) observed this feature in several centrolenids, suggesting its presence as a synapomorphy for glass frogs. However, as more researchers pay attention to this structure, more reports emerge. For instance, recently, Nascimento et al. (2021) reported the presence of vacuities in tadpoles of *Lithodytes lineatus* and in several species of the *Leptodactylus pentadactylus* species group; Dias and Pie (2021) reported them in the larvae of *S. v-signatus* and suggested it as synapomorphy for the *S. perpusillus* species group. These findings suggest that vacuities are more widely distributed within anurans than previously imagined. We observed vacuities in all examined *Atelopus* and also in other unrelated bufonids, such as *Ansonia*, *Incilius*, and *Werneria*. Current optimization suggests that the presence of vacuities in *Atelopus* is a synapomorphy. We also predict that, as more taxa are examined, the presence of vacuities will also optimize as a synapomorphies for *Ansonia* and *Werneria*.

The median ridge is highly variable among anuran larvae (e.g., Wassersug 1980; Vera Candioti 2007; Dias et al. 2019a, 2021), but conical median ridge is particularly rare. It has been reported in few taxa, such as the suctorial *Heleophryne natalensis* (Wassersug and Heyer 1988), and was present in all *Atelopus* examined, although with ambiguous optimization regarding bufonids.

Lack of pustulations and papillae in the buccal floor and roof is not common in anurans and often associated with endotrophic development (Wassersug and Duellman 1984; Romero-Carvajal et al. 2023). Nevertheless, feeding tadpoles may also present a reduction or lack these features, as in the case of oophagous (e.g., Vera Candioti et al. 2021) and macrophagous (e.g., Dias et al. 2019b, 2023) tadpoles. The diet of *Atelopus* larvae is poorly unknown—as that of most species (Altig et al. 2007)—but some elements of their anatomy may suggest some degree of macrophagy; the secretory tissues involved in filtering particles are reduced, they lack several papillae and pustulation, lingual papillae are absent, and the presence shortened intestines (PHD, personal observation). In captivity, the larvae of *Atelopus flavescent* were reported to feed on algae (Gawor et al. 2012), but fish food was also supplemented. Both captivity and field observation as well as detail study of trophic ecology are necessary to better understand what these tadpoles eat.

We observed a single pair of infralabial papillae in *Atelopus* larvae. This condition differs of that observed in tadpoles of Odontophrynidae that usually present two pairs of infralabial papillae (e.g., Nascimento et al. 2013; Dias 2020). Tadpoles of other closely related lineages, such as centrolenids and leptodactylids, also present two pairs of infralabial papillae, (e.g., Wassersug and Heyer 1988; Vera Candioti et al. 2007; Rada et al. 2019; Dias et al. 2020; Nascimento et al. 2020). Dubeux et al. (2023) suggested that the presence of two infralabial papillae could represent a synapomorphy of Bufonidae, and we provide additional evidence for that hypothesis.

Lingual papillae are also present in most anuran larvae, with some few exceptions (e.g., micohylids and several Dendropsophini; Vera Candioti 2007; Dias et al. 2023). All *Atelopus* lack lingual papillae, as well as the endotrophic larvae of *Frostius* (Dubeux et al. 2023) and the direct developer *Osornophryne* (Romero-Carvajal et al. 2023). The optimization of this character state is ambiguous, but it is interesting noting that absence of it in *Frostius* and in *Osornophryne* is probably related to endotrophic development, while *Atelopus* retained a plesiomorphic state or lost those papillae independently is an interesting evo-devo question.

### Convergent evolution in gastromyzophorous and suctorial tadpoles

Convergent evolution is the independent evolution of homoplastic character states in different lineages, usually in association with similar selective pressures (Losos et al. 1998; Losos 2011). Gastromyzophorous tadpoles evolve independently at least eight times in anurans, although highly concentrated in two clades, bufonids and ranids, with one instance in hylids. Gastromyzophorous tadpoles have been reported in *Amolops*, *Huia*, *Meristogenys*, *Sumaterana*, *Wijayarana*, and *Rana sauteri*—(e.g. Kuramoto et al. 1984; Arifin et al. 2021); in three species of the *Rhinella veraguensis* group (*R. chrysophora*, *R. quechua*, and *R. veraguensis*), in *Sabahphrynus maculatus*, in *Adenomus kandianus*, and in *Bufo aspinius* (Rao and Yang 1994; Matsui et al. 2007; Aguayo et al. 2009; Meegaskumbura et al. 2015); finally, the hylid *Phyllodytes gyrinaethes* is also gastromyzophorous (Peixoto et al. 2003; Vera Candioti et al. 2017).

**Fig. 12:**
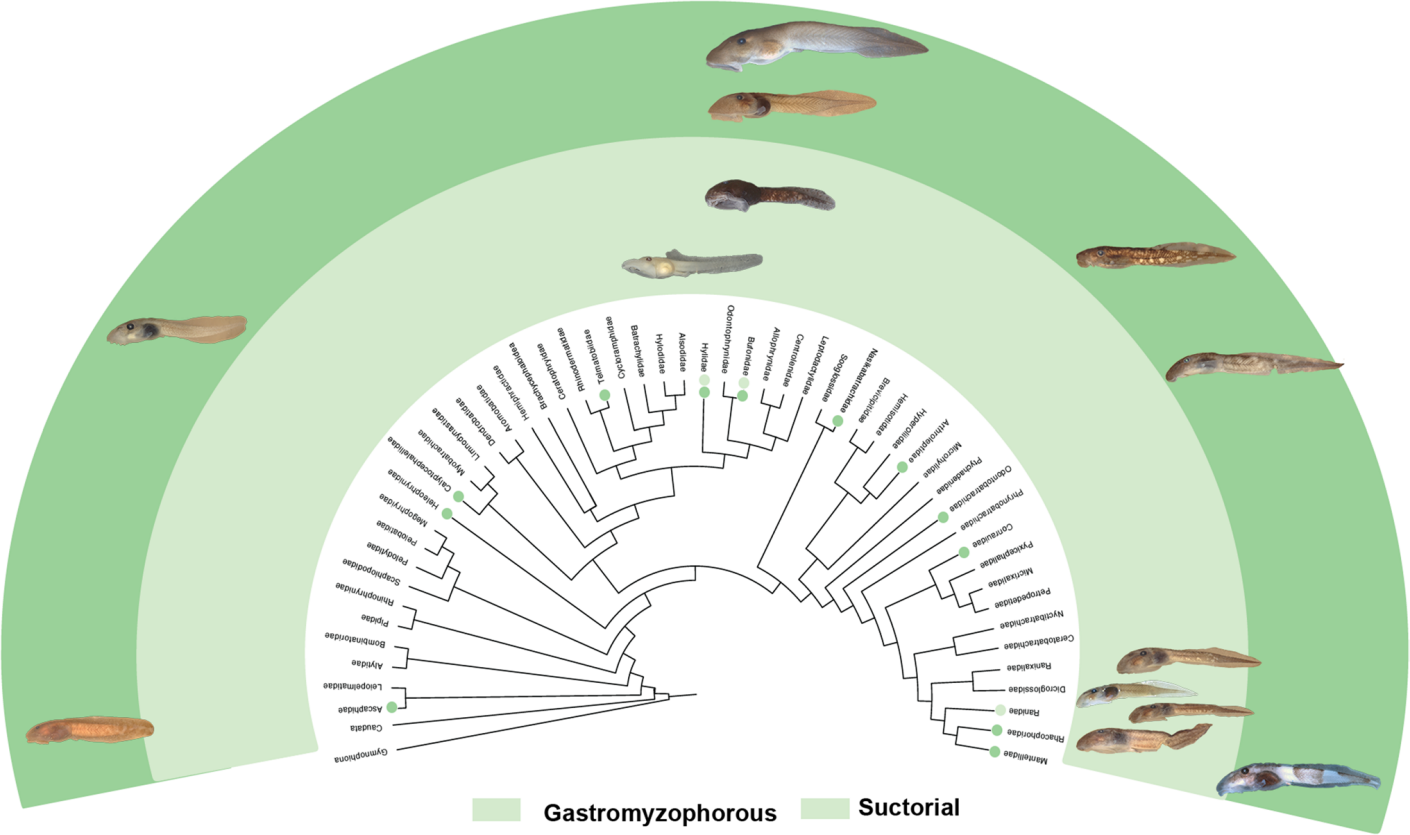
Gastromyzophorous and suctorial larvae evolved independently several times within anurans. The phylogenetic hypothesis of Jetz and Pyron (2018) showing the families in which these tadpoles have evolved

**Fig. 13:**
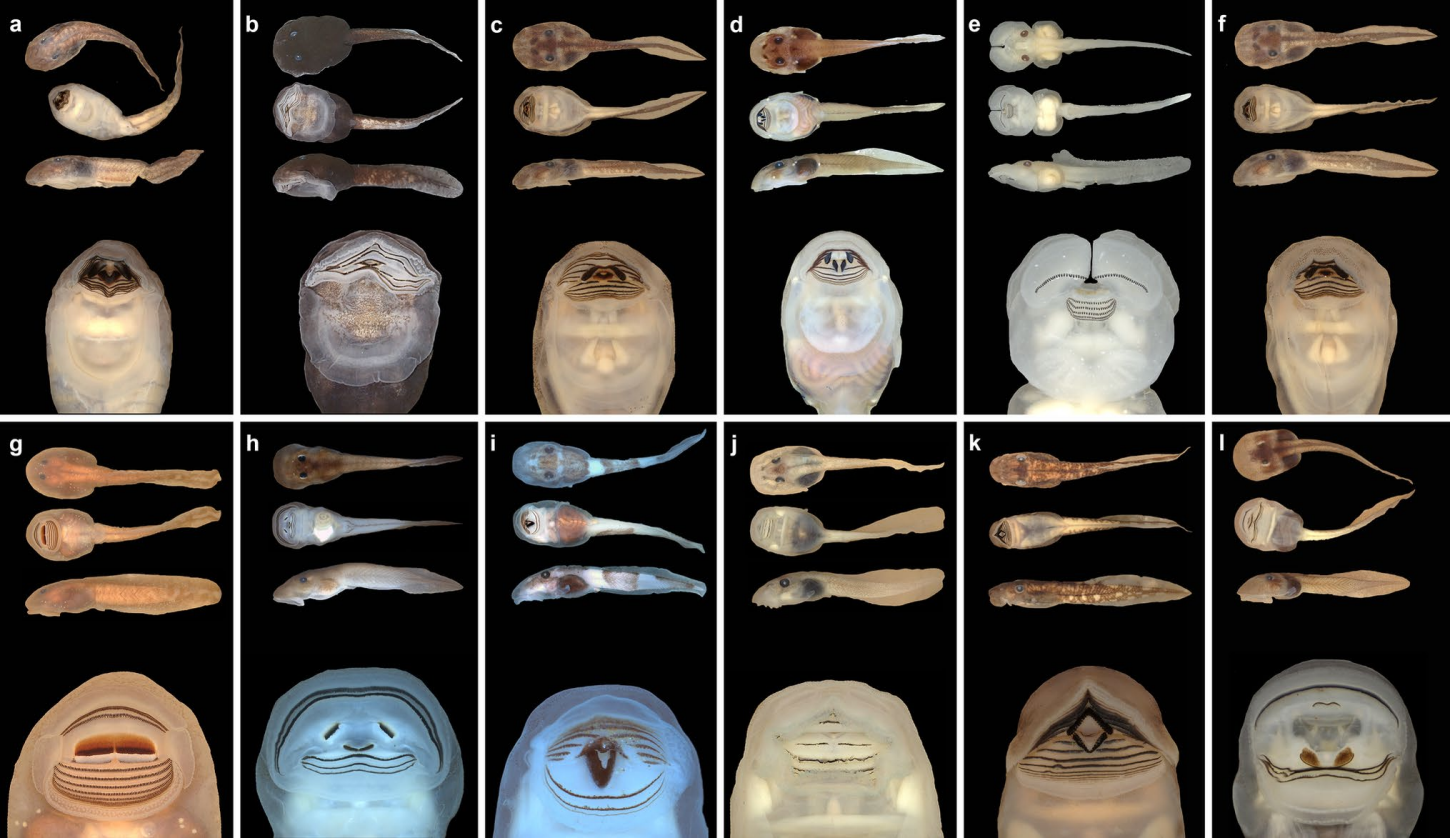
Morphology of gastromyzophorous (**a**–**f**) and suctorial (**g**–**l**) tadpoles. Dorsal views and details of the oral disc; dorsal, ventral, and lateral views and detail of the oral disc of *Amolops cremnobatus* (**a**), *Atelopus subornatus* (**b**), *Mesristogenys jerboa* (**c**), *Huia cavitympanum* (**d**) *Phyllodytes gyrinaethes* (**e**), *Sumaterana dabulescens* (**f**), *Ascaphus truei* (**g**), *Ansonia hanitschi* (**h**), *Boophis schubae* (**i**), *Heleophryne hewitti* (**j**), *Astylosternus robustus* (**k**), and *Werneria mertensiana* (**l**)

Notwithstanding, the development of a belly sucker was not the only solution provided by natural selection to enable tadpoles to adhere to the substrate in fast-flowing waters; suctorial tadpoles also evolved in several lineages (Fig. 12), such as *Ansonia*, *Nasikabatrachus*, *Odontobatrachus*, and many hylids. Gastromyzophorous and suctorial tadpoles, in general, have convergent phenotypic traits, such as enlarged oral discs, depressed bodies with extended and broad snouts, robust and well-keratinized jaw sheaths, strong tails with reduced tail fins, dorsal eyes (Fig. 13), and several modifications in the musculoskeletal system (Gan et al. 2015; Vera Candioti et al. in press).

The buccopharyngeal cavity of gastromyzophorous and suctorial tadpoles also evolved convergently. The buccopharyngeal cavity has been described for few taxa: *Amietia ruwenzorica* (Viertel et al. 2012) *Ansonia longidigita*, *Ansonia hanitschi*, *Ansonia minuta* (Inger 1985; Haas and Das 2008), *Ascaphus truei* (Wassersug 1980), *Heleophryne natalensis* (Wassersug and Heyer 1988), *Huia cavitympanum*, *Meristogenys phaeomerus*, *Meristogenys poecilus*, *Meristogenys kinabaluensis* (Inger 1985), *Nasikabatrachus sahyadrensis* (Raj et al. 2012), and *Rhinella quechua* (Aguayo et al. 2009).

A different combination of the characters described here for *Atelopus* is present in several of these species (Table 2; Fig. 13). For instance, the presence of a developed element in the prenarial arena is almost invariable within gastromyzophorous and suctorial larvae; similarly, vacuities are present in the many of taxa, suggesting an adaptive value in these traits. Unfortunately, for many of these characters, there are no robust hypotheses about their function and how they might increase fitness in torrent environments is still obscure.

**Table 2:** Comparative buccopharyngeal morphology among gastromyzophorous and suctorial larvae

Taxa/character	Prenarial arena element	Narial vacuities	Median ridge	Papillae buccal roof	Lingual papillae	Reference
*Amietia*	Present	Absent	Triangular	Abundant	Present	Viertel et al. (2012)
*Ansonia*	Present	Present	Conical	Absent	Absent	Inger (1985); Haas and Das (2008); this study
*Ascaphus*	Absent	Present	Absent	Absent	Present	Wassersug (1980); this study
*Astilosternus*	Absent	Absent	Absent	Reduced	Absent	This study
*Atelopus*	Present	Present	Conical	Absent	Absent	This study
*Boophis*	Present	Present	Conical	Absent	Absent	This study
*Corythomantis*	Present	Present	Triangular	Reduced	Present	Oliveira et al. (2017)
*Heleophryne*	Present	Present	Conical	Absent	Present	Wassersug and Heyer (1988)
*Hyloscirtus*	Present	Present	Conical	Reduced	Present	Aguilar et al. (2007)
*Huia*	Absent	Absent				Inger (1985)
*Megastomatohyla*	Present	?	Conical	Reduced	Absent	Wassersug (1980)
*Meristogenys*	Absent	Present	Conical	Reduced	Absent	Inger (1985); this study
*Nasikabatrachus*	Present	Absent				Raj et al. (2012)
*Odontobatrachus*	Absent	Present	Absent	Reduced	Absent	This study
*Rhinella*	Present	Absent	Present	Reduced	Absent	Aguayo et al. (2009)
*Telmatobius*	Absent	Absent	Triangular	Abundant	Present	Aguilar et al. (2007)

Nevertheless, the fact that some species described as suctorial (e.g., *Amietia ruwenzorica*) differ phenotypically from that pattern (see Viertel et al. 2012), resembling rheophilous larvae (e.g., Montesinos et al. 2022, 2023), suggests that internal morphology characters should also be included in the studies of ecomorphological guilds of anurans. Moreover, it also suggests that the current structure of ecomorphological guilds might hide ecological, functional, and morphological diversity.

## Conclusion and remarks

The buccopharyngeal cavity provided additional information to understand the taxonomy and the evolution of *Atelopus*. Our study can be added to the growing list of studies about larval morphology of previously poorly known groups in the last 20 years; one important conclusion from those studies is that as more species are investigated, novel and astonishing new morphologies are discovered (e.g., Haas et al. 2006, 2014; Grosjean et al. 2011; Rowley et al. 2012; Vera Candioti et al. 2017, 2021; Dias 2020; Dias et al. 2023). Also, tadpoles have been proven excellent model organism to study evolutionary phenomena; for instance, convergent evolution has been constantly reported in tadpoles of different lineages (e.g., Rada et al. 2019; Grosjean and Preininger 2020) demonstrating how they can be used to better understand the independent evolution of similar phenotypes. This highlights the importance of training new generations of morphologists and evolutionary biologists interested in tadpoles.

## Appendix. Examined material

All the material used in the present study is housed at American Museum of Natural History (AMNH), Amphibians’ Collection, Universidade Federal do Estado do Rio de Janeiro (UNIRIO), Eugenio Izecksohn, deposited at Universidade Federal Rural do Rio de Janeiro (EI), Herpetological Collection of the Universidad del Magdalena (CBUMAG), Instituto de Ciencias Naturales, Universidad Nacional de Colombia (ICN), Museo de Zoología de la Pontificia Universidad Católica del Ecuador (QCAZ), Museu de História Natural, Universidade Federal de Alagoas (MUFAL), Universidad de Costa Rica (UCR), University of Michigan Museum of Zoology (UMMZ), Zoologisches Forschungsinstitut und Museum Alexander Koenig (ZFMK), Zoological Museum Hamburg (ZMH), Zoologisches Museum Berlin (ZMB), and Zoologische Staatssammlung München (ZSM).

**Table:**

Taxa	Buccopharyngeal cavity
*Amazophrynella minuta*	ICN 54915
*Anaxyrus americanus*	UMMZ 139255
*Ansonia hanitschi*	ZMH A08803
*Atelopus balios*	QCAZ 2670
*Atelopus carrikeri*	CBUMAG 0892
*Atelopus nahumae*	ICN 33202
*Atelopus nanay*	QCAZ 3672
*Atelopus subornatus*	ICN 31435
*Barbarophryne brongersmai*	ZMH 12162
*Bufo bufo*	Viertel 1982
*Dendrophryniscus brevipollicatus*	UNIRIO 3394
*Duttaphrynus melanostictus*	ZMH 13591
*Frostius pernambucensis*	EI 7253
*Incilius coniferus*	UCR 18999
*Ingerophrynus divergens*	Inger 1985
*Melanophryniscus klappenbachi*	Baldo et al. 2014
*Osornophryne occidentalis*	Romero-Carvajal et al. 2023
*Peltophryne peltocephala*	AMNH 38451
*Poyntonophrynus fenoulheti*	Lambris 1994
*Rentapia hosii*	Inger 1985
*Rhaebo glaberrimus*	ICN 49629
*Rhinella marina*	ICN 53853
*Schismaderma carens*	Lambris 1994; Viertel and Channing 2017
*Sclerophrys maculatus*	ZMH 11955
*Vandijkophrynus gariepensis*	Lambris 1994
*Werneria mertensiana*	ZMB 79695
Other taxa	
*Amolops cremnobatus*	ZFMK 95596
*Ascaphus truei*	ZFMK 44444
*Astylosternus robustus*	ZMB 82040
*Boophis schubae*	ZSM 817–2004
*Heleophryne hewitti*	ZMB 74986
*Huia cavitympanum*	ZMH13441
*Macrogenioglottus alipioi*	MUFAL 10811
*Meristogenys jerboa*	ZMH10164
*Odontobatrachus fouta*	ZMB 88109
*Odontophrynus cultripes*	UFMG 937
*Phyllodytes gyrinaethes*	MUFAL
*Proceratophrys appendiculata*	UNIRIO 4036
*Sumaterana dabulescens*	ZMH12654
*Werneria mertensiana*	ZMB 79695
